# Living in the dark: Bat caves as hotspots of fungal diversity

**DOI:** 10.1371/journal.pone.0243494

**Published:** 2020-12-04

**Authors:** Aline O. B. Cunha, Jadson D. P. Bezerra, Thays G. L. Oliveira, Eder Barbier, Enrico Bernard, Alexandre R. Machado, Cristina M. Souza-Motta

**Affiliations:** 1 Departamento de Micologia Prof. Chaves Batista, Centro de Biociências, Universidade Federal de Pernambuco, Recife, Brazil; 2 Setor de Micologia, Departamento de Biociências e Tecnologia, Instituto de Patologia Tropical e Saúde Pública, Universidade Federal de Goiás, Goiânia, Brazil; 3 Laboratório de Ciência Aplicada à Conservação da Biodiversidade, Departamento de Zoologia, Centro de Biociências, Universidade Federal de Pernambuco, Recife, Brazil; Universita degli Studi di Pisa, ITALY

## Abstract

Bat caves are very special roosts that harbour thousands of bats of one or more species. Such sites may hold an incredible “dark fungal diversity” which is still underestimated. We explored the culturable fungal richness in the air, on bats, and in the guano in a bat cave in Brazil’s Caatinga dry forest. Fungal abundance was 683 colony-forming units (CFU) in the guano, 673 CFU in the air, and 105 CFU on the bats. Based on morphological and phylogenetic analysis of ITS, LSU, and *TUB2* sequences, fungal isolates of 59 taxa belonging to 37 genera in the phyla Ascomycota (28 genera, including *Aspergillus*, *Penicillium*, *Cladosporium*, and *Talaromyces*), Basidiomycota (eight genera, including *Rhodotorula* and *Schizophyllum*), and Mucoromycota (only *Rhizopus*) were identified. The fungal richness in the air was 23 taxa (especially *Aspergillus* taxa), mainly found at 15 m and 45 m from the cave entrance; on the bodies of bats it was 36 taxa (mainly *Aspergillus* taxa), especially on their wing membranes (21 taxa, nine of which were exclusively found in this microhabitat); and in guano 10 fungal taxa (especially *Aspergillus* and *Penicillium*) were found. The fungal richness associated with guano (fresh and non-fresh) was similar from bats with different eating habits (insectivorous, frugivorous, and haematophagous). Sampling effort was not sufficient to reveal the total fungal taxa richness estimated. Eight (21.6%) of the 37 genera and 17 (53.1%) of the 32 identified fungal species are reported for the first time in caves. Our results highlight bat caves in Brazil as hotspots of fungal diversity, emphasizing the need to protect such special roosts.

## Introduction

Bat caves are special habitats which harbour large bat communities that frequently surpass 100,000 individuals [e.g. 1, 2]. Such caves provide specific microclimatic conditions that favour many species of bats to use these underground environments [2–4]. As a consequence of such large numbers of individuals together, bat caves usually contain large deposits of bat guano, supporting rich and complex cave biota [5]. Cave biota are frequently identified as being highly diverse and specialised (e.g. troglobiont, eutroglophile, subtroglophile, and trogloxene) [6, 7], and are known to show extreme endemism; a myriad of unknown species are often discovered [8–10]. This is also true for fungi.

In recent years, fungal diversity associated with bats and caves has received much attention because several bat species have been affected by the white-nose syndrome caused by an ascomycetous fungal species (*Pseudogymnoascus destructans* (Blehert & Gargas) Minnis & D.L. Lindner) [11, 12]. Although nearly 2,000 fungal species have been recorded from caves and similar environments [5, 13–18], our knowledge of their richness and diversity in these environments is far from complete. A recent study from karst caves in China described 20 new fungal species belonging to 18 genera in Ascomycota [16], concluding that the sampled caves harbour a high fungal diversity [see 16, 17].

A better understanding of the origin and evolution of fungal diversity in caves is still a scientific challenge. Recently, Zhang et al. [7] studied the divergence time of suspected troglobitic fungi in karst caves in China and concluded that this mycobiome came from other environments. Other studies have indicated that several fungal species discovered from caves, on bats, and in guano are fungi known from other habitats, and have suggested that external vectors from an epigean environment (trogloxene animals, for example) may have a role in shaping the fungal community in caves [11, 16, 19, 20]. In addition to the effect of air currents, water movement, and visitors [7, 11, 16, 21], bats are probably the most important vector in the dispersal of fungal species in caves [22, 23].

Brazil harbours 181 bat species [24] and at least 72 of these are reported to use caves [25, 26]. However, the fungal diversity of caves in Brazil is still poorly explored, and needs to be studied to contribute to Brazilian and global fungal estimations. Studies conducted in Brazilian caves have focused on verifying the environmental quality for tourism proposals, and therefore, their search was directed towards finding pathogenic fungal species [21, 27, 28]. The first report of fungal species from caves in Brazil (in the Amazon forest) was published by Castrillón et al. [29], who isolated fungi from soils and identified these as belonging to eight ascomycetous genera, including a rare dermatophyte, *Microsporum amazonicum* (currently *Arthroderma amazonicum* (Moraes, Borelli & Feo) Y. Gräser & de Hoog), and several other isolates without any taxonomic identification rank (e.g. yeasts and unknown fungi). Later, Taylor et al. [21, 24] studied cave fungi from the air, guano, and various sediments and found species mainly belonging to genera such as *Aspergillus* and *Penicillium* of the phylum Ascomycota. As a consequence of the possible infection of eight biologists by *Histoplasma capsulatum* Darling after an expedition to a cave, Rocha-Silva et al. [28] highlighted the importance of verifying the presence of this species before authorisation for public visitation. However, Paula et al. [30, 31] demonstrated the high cellulolytic activities of fungal isolates (including *Aspergillus*, *Penicillium*, and *Talaromyces*) from the soil of a cave, pointing to some biotechnological potential. To the best of our knowledge, there are no studies on the fungal species richness from bat caves in Brazil (including fungi that are airborne, found on bats, and in the guano), particularly in the Brazilian tropical dry forest.

The Brazilian territory occupies more than 53% of South America and is recognised as one of the most megadiverse countries in the world [32, 33]. The Caatinga, located in North-eastern Brazil, is part of the so-called Dry Diagonal of South America and is recognised as one of the most diverse dry forests globally [34]. Furthermore, most known bat caves in Brazil are located in this region [2]. The fauna, flora, and fungi of the Caatinga dry forest have recently received a lot of attention as a result of several studies describing new taxa, ecological patterns, and behaviour of species [35–37], and because of the anthropic pressure and plans for conservation of natural areas [38, 39]. Nevertheless, the Caatinga’s fungal diversity is better known in association with soil, plants, and as decomposers [40, 41], but the mycodiversity associated with bats, which comprise approximately 96 species in this biogeographic region [2, 42], is still unexplored.

Given the poorly known and studied fungal diversity associated with bats, their habitats, and substrates, and considering that this is even more critical in megadiverse and continental-sized countries like Brazil, in this study we 1) assessed the fungal species richness of airborne fungi from a bat cave in a poorly-sampled part of the country, 2) analysed the fungal presence in three microhabitats on the body of bats (oral cavity, fur, and wing membrane), and 3) detected fungi associated with guano (fresh and non-fresh) from bats with different eating habits (insectivorous, frugivorous, and haematophagous).

## Material and methods

### Study area

The Catimbau National Park (IUCN Category II) is located in the state of Pernambuco, North-eastern Brazil (8°24′00″ and 8°36′35″ S; 37°0′30″ and 37°1′40″ W) and it is part of the Caatinga domain—the largest, most species-rich tropical dry forest region in South America [37, 43]. Occupying 62,292 ha, the Park falls within the administrative boundaries of three municipalities (Buíque, Ibimirim, and Tupanatinga) and the vegetation found here is a mosaic of dry forest and shrubs, including xeric and non-xeric species [43, 44]. The region’s climate is classified as hot semiarid (Bsh, according to the Köppen-Geiger classification), with an annual mean temperature of 23°C and annual mean precipitation ranging from 486–975 mm, with rainfall being concentrated between April and June [45]. However, as in other regions of the Caatinga, past rainfall has been irregular and there may be long periods of drought.

**Fig 1:**
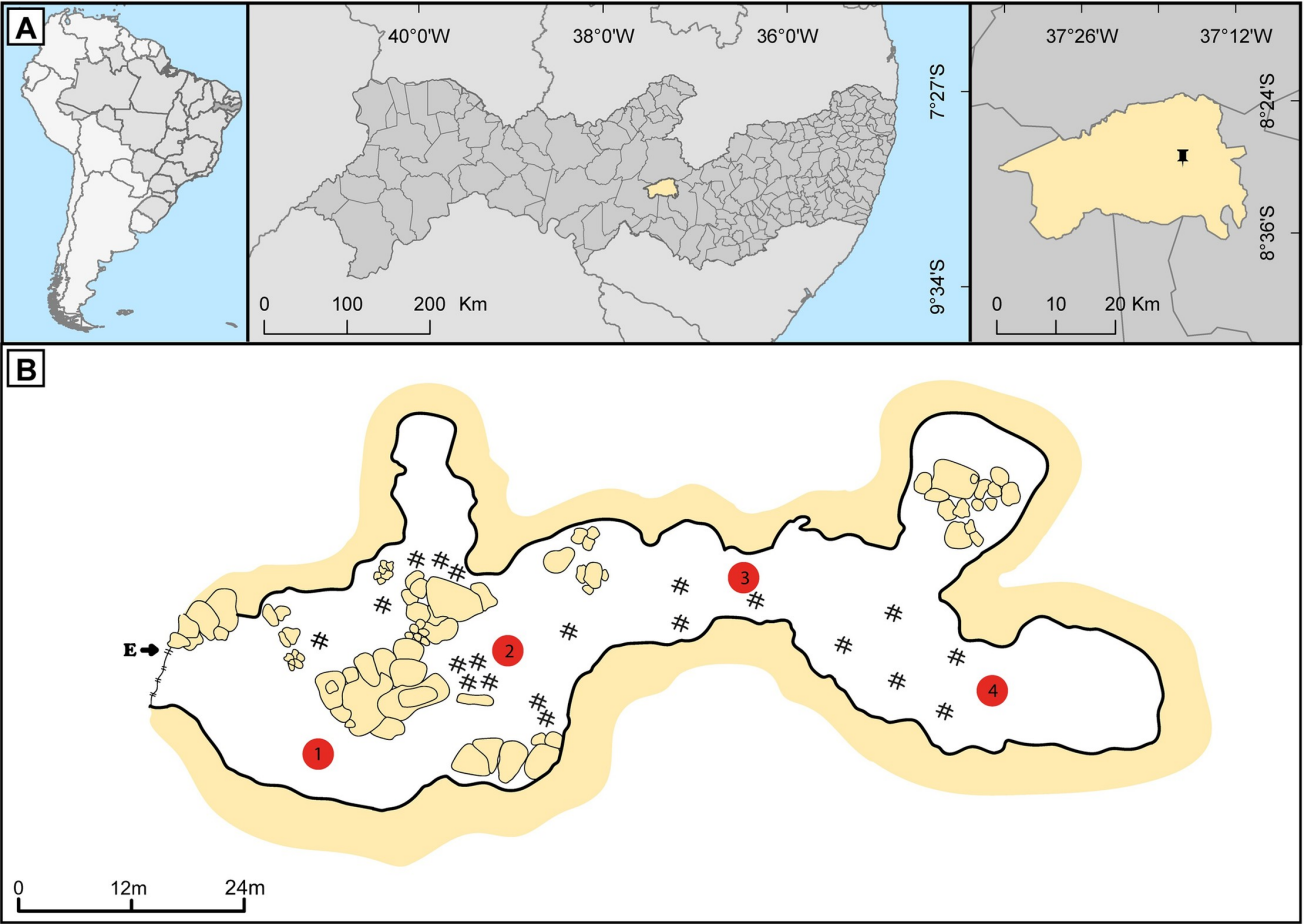
*Meu Rei* bat cave. A. The geographical location of the cave at the Parque Nacional do Catimbau [Catimbau National Park], Tupanatinga municipality, Pernambuco state, Brazil. B. Cave sketch showing the entrance (E), presence of guano (#), and the four sampling points (1, 2, 3, and 4). The cave sketch was based on one drawn by the CECAV/ICMBio-MMA, Brazil.

**Fig 2:**
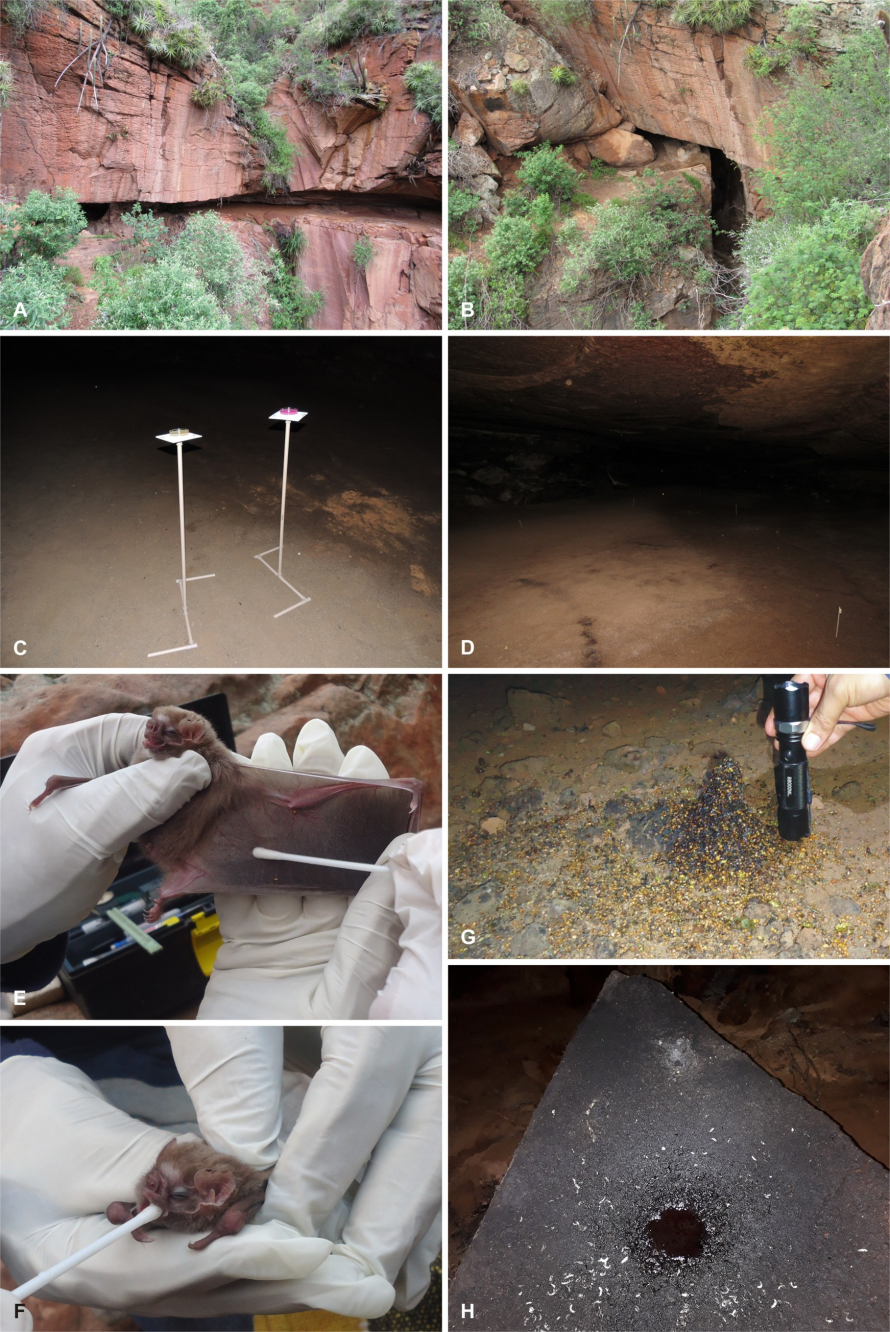
*Meu Rei* bat cave at the Parque Nacional do Catimbau [Catimbau National Park], Pernambuco state, Brazil. A. Outside frontal view of the cave. B. Detail of the cave entrance. C. Petri dishes that were used to sample airborne fungi at sampling point 2 (cave floor comprised soil and guano). D. Sampling point 4 showing cave floor covered with bat guano. E. Sample collection of fungi from the wing membrane of *Diphylla ecaudata*. F. Sample collection of fungi from the oral cavity of *D*. *ecaudata*. G. Frugivorous guano of *Carollia perspicillata* (some seeds can be seen). H. Haematophagous guano of *D*. *ecaudata* on a rock (fungal mycelia were observed colonising the guano). Photos were taken by A.O.B. Cunha, E. Barbier, and N.T. Pimentel.

*Meu Rei* is a sandstone cave located in the Catimbau National Park (in Tupanatinga municipality), about 162 m long, with a single entrance, and divided into four main chambers [4, 46] (Figs 1 and 2). This cave is considered a bat cave owing to the large colony using it as a shelter. The colony sometimes reaches ~120,000 individuals, mainly consisting of the insectivorous bat *Pteronotus gymnonotus* (family Mormoopidae). However, there is a large fluctuation in the size of bat populations throughout the year [2]. In addition to *P*. *gymnonotus*, at least nine other bat species belonging to the Natalidae and Phyllostomidae families—including frugivorous, sanguinivorous, nectarivorous, and omnivorous species—use this cave for shelter [2, 47]. Measurements in the central and deepest portions of the cave recorded an average ambient temperature of 25°C and 28°C and relative humidity of 80% and 87%, respectively [47]. Fieldwork was authorised by licences from MMA/ICMBio (SISBIO numbers 43816–1 and 43816–2) and the Ethics Committee on Animal Care–UFPE (number 23076.027916/2015-13) and was conducted in September 2017. Bat captures and handling methods followed the guidelines of the American Society of Mammalogists [48].

### Sampling stations

In order to verify the diversity of fungi along the linear development of the cavity, we sampled at four points: 15 m (point 1), 45 m (point 2), 75 m (point 3), and 135 m (point 4) from the cave entrance (Figs 1 and 2). Samples to evaluate airborne fungi, and whenever possible, guano samples were collected at these four pre-defined points. These points were defined not only because they represent a gradient of light, airflow, temperature, humidity, and entrance distance, but also because they harbour bat colonies of different species, as follow: Point 1 = *Diphylla ecaudata*; Point 2 = *Carollia perspicillata* and *Glossophaga soricina*; Point 3 = *Lonchorhina aurita* and *Tonatia bidens*; and Point 4 = *Anoura geoffroyi*, *Natalus macrourus*, *Pteronotus gymnonotus*, and *P*. *personatus*. *Desmodus rotundus* sporadically used point 1 as shelter but they were not observed when we were sampling.

### Airborne fungi sampling and isolation

Airborne fungi sampling was performed during the night (~ 7:00 pm) after the bats left the cave for foraging (Fig 2C). The gravity settling method using 90 mm Petri dishes with Dichloran-Rose Bengal Chloramphenicol (DRBC) agar and Brain Heart Infusion (BHI) agar culture media was used for the isolation of fungi from the cave air [see 16, 21]. Six Petri dishes (three for each culture medium) were randomly distributed at each point (see Sampling stations section). At each sampling point, the Petri dishes were positioned 1 m above the ground and opened simultaneously. These were left open for 20 min, after which the plates were closed, labelled, and taken to the laboratory. The plates were incubated at 28°C for at least 7 days in the dark. Selected fungal colonies were sub-cultured using Sabouraud dextrose agar medium plus chloramphenicol (100 mg/L) to restrict bacterial growth.

### Capturing bats, sampling, and fungal isolation

Bats were captured inside the cave between 4:00 pm and 4:30 pm, using a hand net. Three individuals of *C*. *perspicillata* (frugivore) and four of *D*. *ecaudata* (sanguivore) were captured during this study and released once samples were taken (Fig 2E and 2F). During the fieldwork (September 2017), there were approximately only 136 bats in the cave [2], making it impossible for us to capture more individuals because they were sheltered in inaccessible places on the cave roof. From each individual, samples were collected from three microhabitats on the bat’s body: oral cavity, fur (belly and back), and wing membrane (ventral and dorsal surface). Samples were collected using sterile swabs that were pre-moistened with sterilised water plus chloramphenicol (0.1 mg/L). These were gently introduced in the oral cavity and were rolled back-and-forth three times across the bat fur and wing membrane. Swabs were then individually placed in sterilised 15 mL conical centrifuge tubes containing water plus chloramphenicol (0.1 mg/L), labelled, stored chilled, and shipped for processing. At the laboratory, conical centrifuge tubes were shaken, and 2 mL of the solution was used to inoculate Petri dishes containing the BHI and Sabouraud dextrose agar media. Petri dishes containing BHI were incubated at 28°C and the plates containing Sabouraud dextrose agar at 37°C, both for at least 7 days in the dark. Fungal growth was observed every day, and all the colonies were isolated and purified using Sabouraud dextrose agar medium supplemented with chloramphenicol (100 mg/L).

### Guano sampling and fungal isolation

Fresh and non-fresh guano were collected from bats with different eating habits: insectivorous (*Pteronotus* spp.), frugivorous (*C*. *perspicillata*), and haematophagous (*D*. *ecaudata*) (Fig 2G–2H). The guano samples from these bat species were chosen because of the individuals’ fidelity to certain places inside the cave, allowing a correct association with the bat and also because of the availability of the guano owing to a reduced number of individuals and bats species during the fieldwork. At the laboratory, 1 g from each guano pile was placed into a 250 mL Erlenmeyer flask containing 9 mL of distilled and sterilised water plus chloramphenicol (0.1 mg/L). Erlenmeyer flasks (9 mL water + guano) were manually shaken and used to make a dilution up to 10^−4^ from which 1 mL was used to inoculate Petri dishes with DRBC agar and Sabouraud dextrose agar plus chloramphenicol (100 mg/L) [see 16, 21]. Petri dishes were incubated at 28°C for at least 7 days in the dark, and representative isolates of the total fungal colonies grown were taken and purified using Sabouraud dextrose agar plus chloramphenicol (100 mg/L).

### Fungal identification

Fungal isolates obtained in this study were first grouped into morphospecies based on macro- and micro-morphological features. Thereafter, molecular analyses were undertaken. Colonies were grown on potato dextrose agar (PDA) or malt extract agar (MEA); after at least 7 days of incubation at 28°C in the dark these were used to perform DNA extraction following the manufacturer’s protocol for the Wizard Genomic DNA Purification Kit. The primers ITS5/ITS4 [49], LR0R/LR5 [50, 51], and Bt2a/Bt2b [52] were used to amplify the ITS rDNA region of 121 filamentous fungi isolates, part of the LSU (D1/D2 domains) region of 28 yeasts isolates, and part of the *TUB2* gene of 15 isolates selected from the other filamentous fungi *Aspergillus*, *Penicillium*, and *Talaromyces*, respectively. The amplifications (PCR analyses) were conducted following the protocols described by Bezerra et al. [53]. The manufacturer’s instructions for the BigDye Terminator Cycle Sequencing Kit v.3.1 were used for the amplicon sequencing using the same primer sets. Sequence assembly and editing were performed using MEGA v.7 [54] and later deposited in GenBank (S1 Table).

Phylogenetic analyses were performed using sequences obtained in this study with reference sequences obtained from the GenBank database and following taxonomic papers for each genus. Selected sequences were aligned with our sequences using the online tool MAFFT v.7 [55] and edited manually using MEGA v.7 [54]. First, to infer a preliminary phylogenetic relationship for the newly generated sequences (ITS, LSU, or *TUB2*) within types of reference material of related fungal species, the DNA sequences were organised by fungal groups (e.g. each genus, genera from the same family/order, or sections in genera such as *Aspergillus* and *Penicillium*) producing 35 sequence alignments, which were analysed independently using the maximum likelihood (ML) analysis. Based on the initial analyses and to show the phylogenetic relationship of the generated sequences, all the ITS, LSU, and *TUB2* alignments were used to construct three individual gene alignments using sequences from GenBank (ITS = 351, LSU = 78, and *TUB2* = 49) and the sequences obtained in this study (ITS = 121, LSU = 26, and *TUB2* = 15) which were analysed using Bayesian inference (BI) analysis. BI and ML analyses were conducted with MrBayes v 3.2.7a [56] on XSEDE and RAxML-HPC BlackBox v 8.2.12 [57], respectively, at the CIPRES Science Gateway [58]. The best nucleotide model for the BI analysis was estimated using the program MrModelTest v.2.3 [59] (ITS and LSU = GTR + I + G, and *TUB2* = HKY + I + G). The GTR + I + G model was used for the ML analysis. BI analysis was conducted with 1 × 10^7^ generations, a burning value of 40%, and chains were sampled every 1000 generations; ML analysis was conducted with 1,000 bootstrap replicates. Values equal or higher than 0.95 BI posterior probability are shown near nodes. Alignments were deposited in TreeBASE (study ID S26796).

### Data analyses

Fungal abundance was considered to be the total number of colonies (colony-forming units, CFU), and the values were subjected to ANOVA using the F test with a probability level of 0.05 and 0.01; for the significant results, the Tukey’s test was performed. Airborne fungi and those in guano and on the body of the bats were evaluated as the total number of CFU and richness of fungal species. Species accumulation curves were determined for the air, bat, and guano, and the total richness (observed) was compared with the estimated richness using the first- and second-order Chao (Chao 1 and 2), the first- and second-order Jackknife (Jackknife 1 and 2), and the Bootstrap estimators to evaluate the sufficiency of the sampling effort using the software Primer version 7.0 (Informer Technologies, Inc., Los Angeles, CA). We conducted a Mann-Whitney *U* test to verify whether the different species of bats showed differences in their fungal composition for the same type of microhabitat (i.e. oral cavity, fur, and wing). In addition, we wanted to test whether there was a difference between the fungal compositions associated with different microhabitats in the bat’s body. In this step, using the Kruskal-Wallis test (*H*) followed by a post-hoc Dunn’s test for multiple paired comparisons, we analysed the data together and separately for *C*. *perspicillata* and *D*. *ecaudata*. In these analyses, we used SigmaPlot version 14.0 (Systat Software, San Jose, CA) with a significance level of *P* ≤ 0.05.

## Results

### General fungal abundance and richness

**Fig 3:**
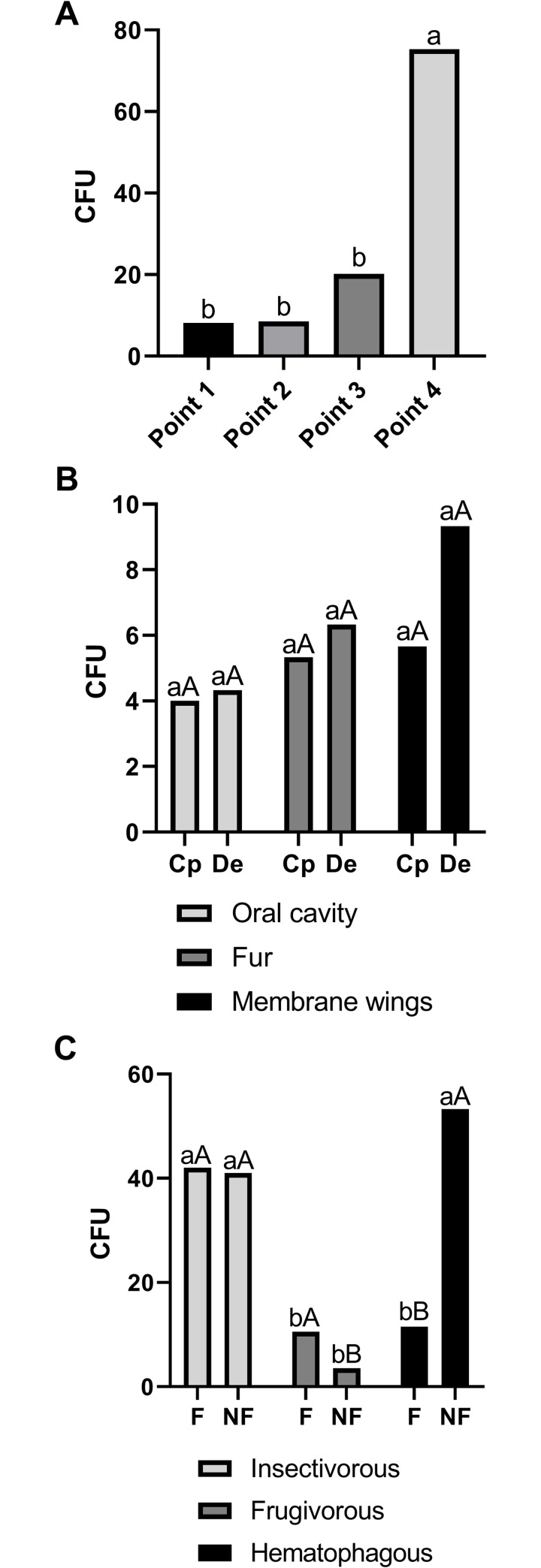
Fungal abundance (CFU). Mean number of colonies (CFU) observed in the samples that were collected to determine airborne fungi (**A**), those on bats (**B**), and those found in the guano (**C**) from the *Meu Rei* bat cave, Catimbau National Park, Caatinga dry forest, Pernambuco state, North-eastern region, Brazil. Cp = *Carollia perspicillata* and De = *Diphylla ecaudata*. F = fresh and NF = non-fresh guano. Different minuscule letter on the bars differ by the Tukey’s test at 5% probability (**A**); lowercase and uppercase letters on the bars differ by the Tukey’s test at 5% probability (**B** and **C**).

**Fig 4:**
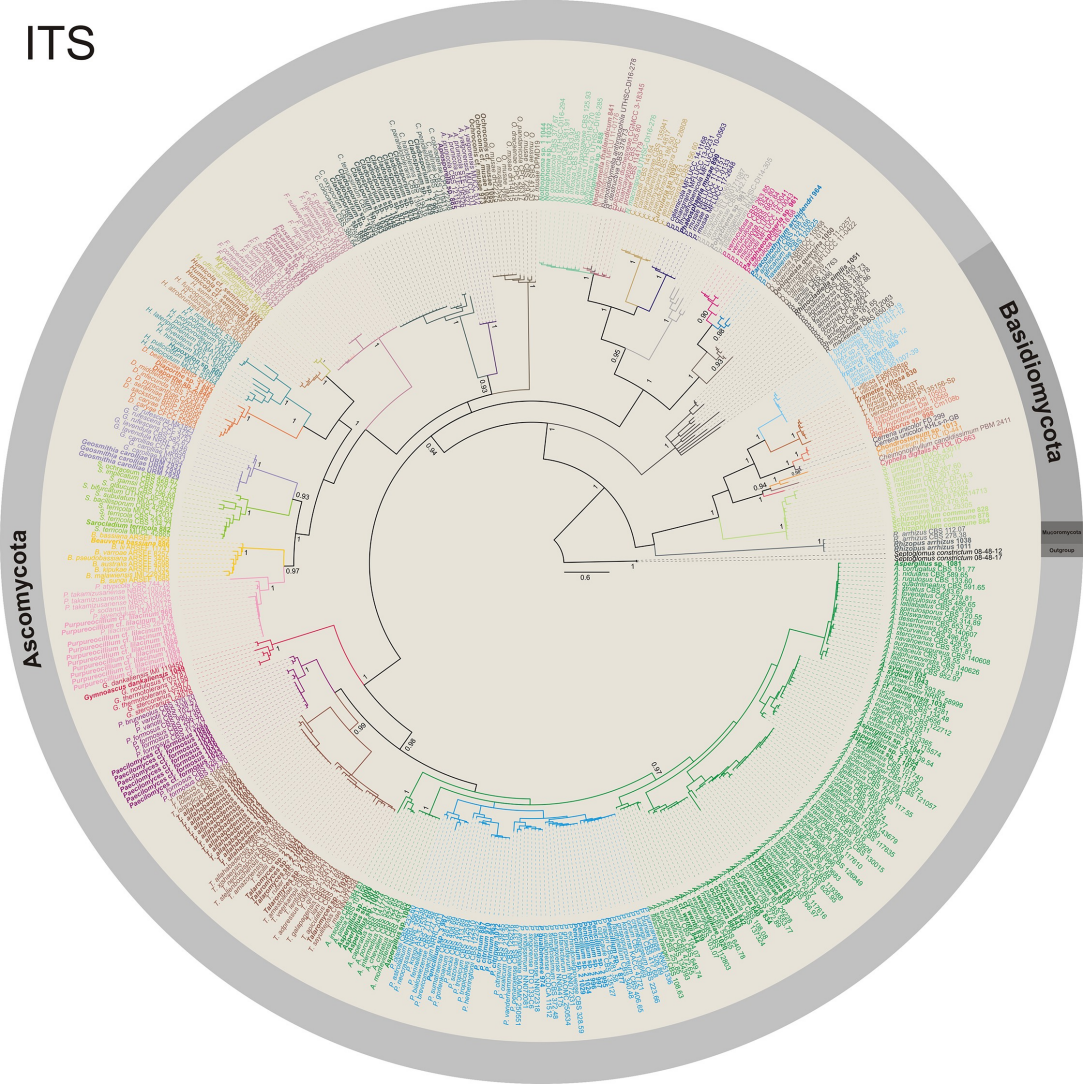
Phylogenetic diversity of filamentous fungi isolates using ITS rDNA sequences. Bayesian inference tree of ITS sequences from fungal isolates of Ascomycota, Basidiomycota, and Mucoromycota found as airborne and isolated from bats and guano in the *Meu Rei* bat cave, Catimbau National Park, Pernambuco state, North-eastern region, Brazil. Isolates obtained in this study are in bold. *Septoglomus constrictum* (08-48-12 and 08-48-17) was used as the outgroup.

**Fig 5:**
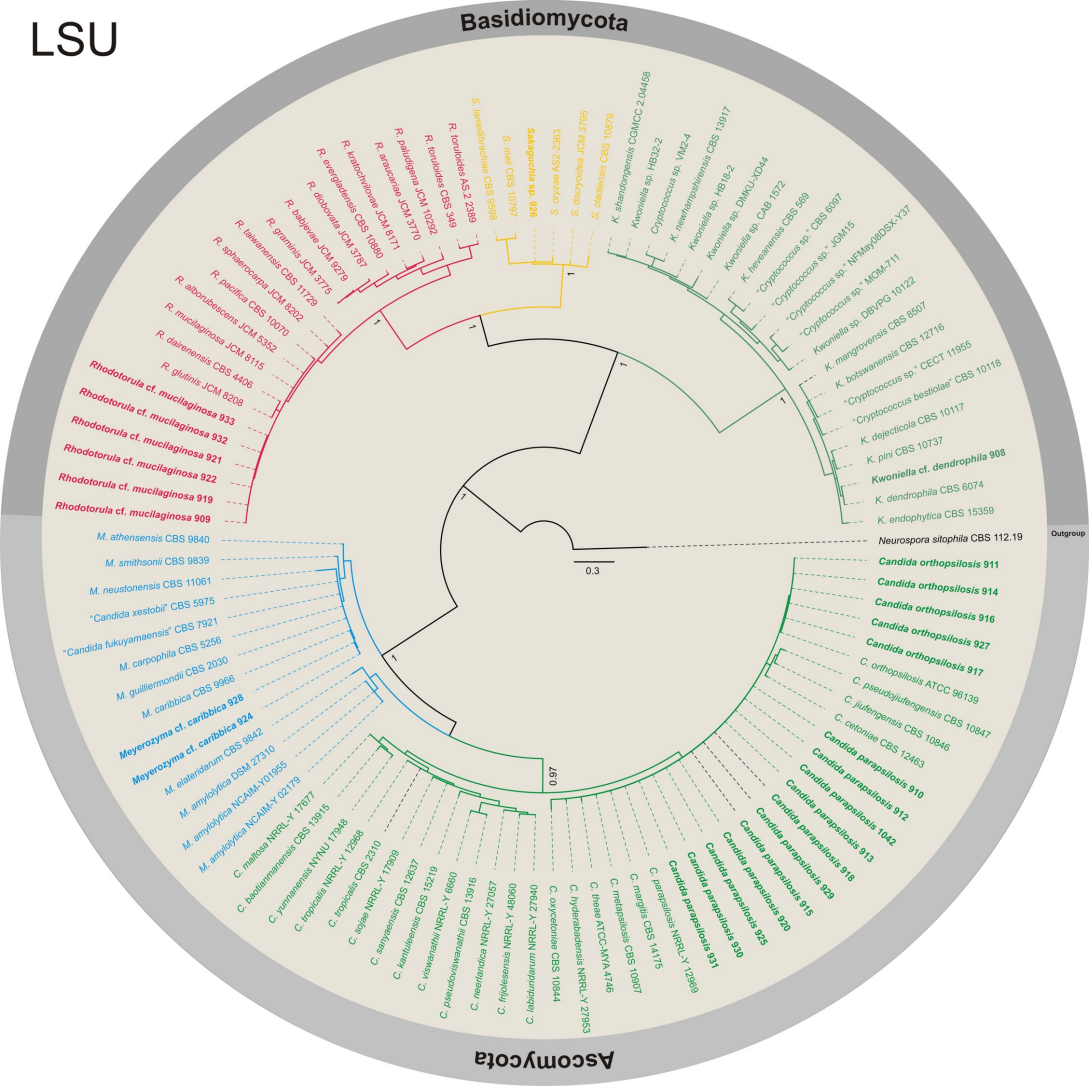
Phylogenetic diversity of yeasts isolates using LSU (D1/D2 domains) rDNA sequences. Bayesian inference tree of LSU sequences from isolates of yeasts from the Ascomycota and Basidiomycota phyla that were isolated from bats in the *Meu Rei* bat cave, Catimbau National Park, Pernambuco state, North-eastern region, Brazil. Isolates obtained in this study are in bold. *Neurospora sitophila* (CBS 112.19) was used as the outgoup.

**Fig 6:**
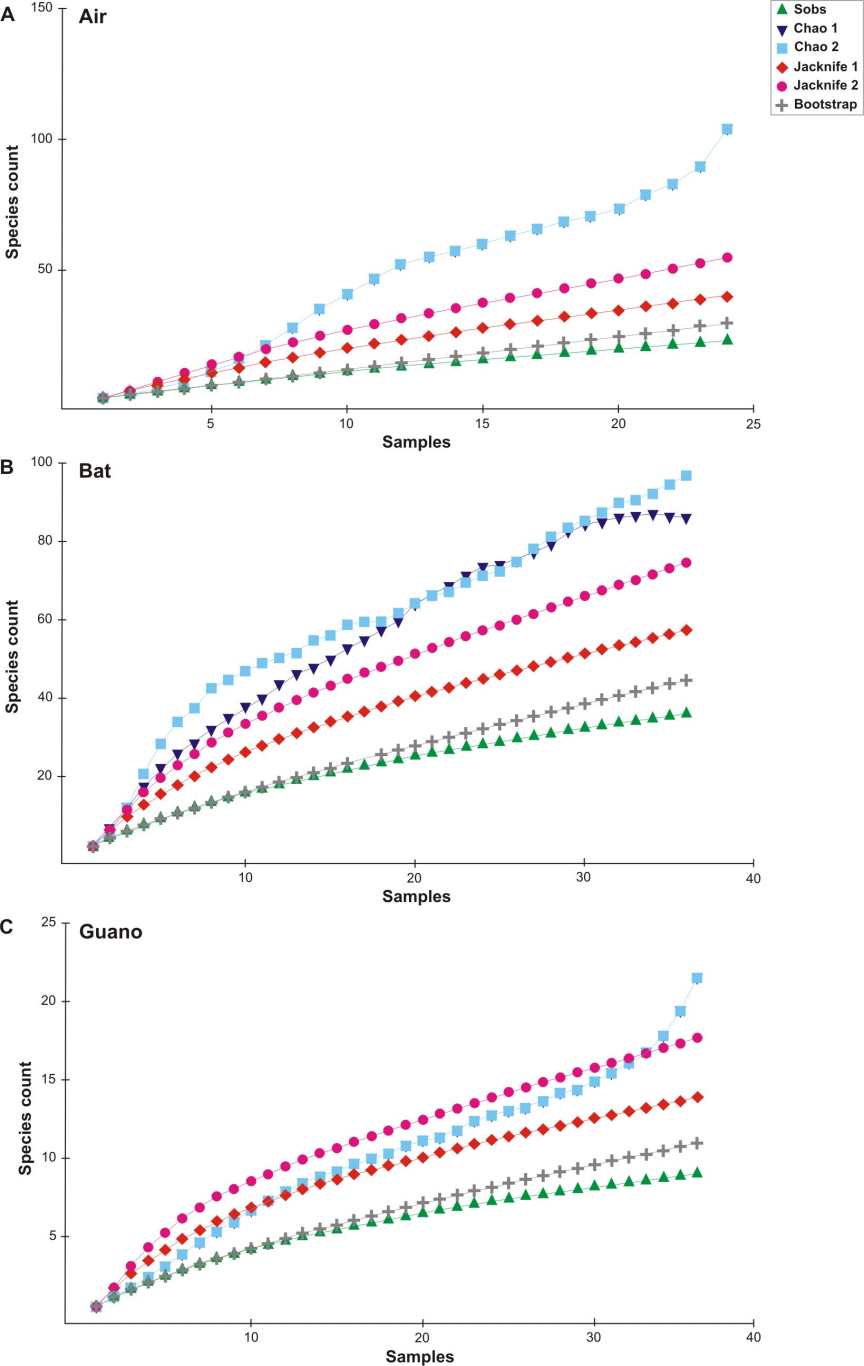
Species accumulation curves. Fungal species observed and estimated (Chao 1, Chao 2, Jackknife 1, Jackknife 2, and Bootstrap) for air (**A**), bats (**B**), and guano (**C**) in the *Meu Rei* bat cave, Catimbau National Park, Caatinga dry forest, Pernambuco state, North-eastern region, Brazil.

Fungal abundance was 683 CFU in the guano, 673 CFU in the air, and 105 CFU on the bats (Fig 3, S2 Table). Fungal isolates were identified using phylogenetic analysis of DNA sequences in 59 taxa. The phylum Ascomycota was the most common, with 50 taxa, followed by Basidiomycota (eight), and Mucoromycota (one) (Figs 4 and 5, Table 1, S1 Fig). In Ascomycota (28 genera), the genus *Aspergillus* presented the largest number of taxa (12), followed by *Penicillium* (five), *Cladosporium* (three), and *Talaromyces* (three). Twenty-four other ascomycetous genera were represented by at least one or two taxa (e.g. *Aplosporella*, *Beauveria*, and *Curvularia*). All genera (eight) in Basidiomycota were represented by one taxon, *Rhodotorula* cf. *mucilaginosa* (isolated from bats), which was the most abundant (six isolates). *Rhizopus arrhizus* (only isolated from bats) was the only representative of Mucoromycota (Table 1). The species accumulation curves of fungal taxa found on air, bats, and guano did not reach a plateau; but, for example, using the Bootstrap estimator, the sampling effort was sufficient to recover 76% (air), 81% (bats), and 81% (guano) of the fungal taxa estimated; in contrast, based on the Chao 2 estimator we found 22% (air), 37.5% (bats), and 42.8% (guano) of the fungal taxa estimated to be found during our survey (Fig 6).

**Table 1:** Fungal taxa richness. List of fungal taxa isolated from air, bat (oral cavity, fur, and wing membrane), and guano in the *Meu Rei* bat cave, Catimbau National Park, Pernambuco state, North-eastern region, Brazil.

Fungi	Record^a^	Air	Bat	Guano
**Ascomycota**				
*Aplosporella* sp.	G	**P**	A	A
*Aspergillus bertholletiae*	S	**P**	A	A
*Aspergillus* cf. *sesamicola*	S	**P**	A	A
*Aspergillus* cf. *tubingensis*		A	A	**P**
*Aspergillus* cf. *wentii*		**P**	**P**	A
*Aspergillus ochraceus*		**P**	A	A
*Aspergillus* sp. 1 section *Nidulantes*		A	**P**	A
*Aspergillus* sp. 2 section *Nigri*		A	**P**	A
*Aspergillus* sp. 3 section *Nigri*		A	**P**	A
*Aspergillus* sp. 4 section *Polypaecilum*		A	A	**P**
*Aspergillus* sp. 5 section *Aspergillus*		A	**P**	A
*Aspergillus sydowii*		**P**	**P**	**P**
*Aspergillus westerdijkiae*	S	A	**P**	A
*Beauveria bassiana*		**P**	A	A
*Candida orthopsilosis*	S	A	**P**	A
*Candida parapsilosis*		A	**P**	A
*Cladosporium* sp. 1 *C*. *sphaerospermum* complex		**P**	A	A
*Cladosporium* sp. 2 *C*. *sphaerospermum* complex		A	**P**	A
*Cladosporium* sp. 3 *C*. *cladosporioides* complex		**P**	**P**	A
*Curvularia* sp.		A	**P**	A
*Deniquelata quercina*	G, S	A	**P**	A
*Diaporthe* sp. 1		**P**	A	A
*Diaporthe* sp. 2		**P**	A	A
*Fusarium* sp. *F*. *fujikuroi* complex		A	**P**	A
*Geosmithia carolliae*	S	A	**P**	A
*Gymnoascus dankaliensis*		A	**P**	A
*Humicola* cf. *seminuda*	S	**P**	A	**P**
*Hypoxylon* sp.		A	**P**	A
*Meyerozyma* cf. *caribbica*	G, S	A	**P**	A
*Myceliophthora* sp.		**P**	A	A
*Neodidymella thailandicum*	G, S	**P**	A	A
*Nothophoma* sp. 1	G	A	**P**	A
*Nothophoma* sp. 2		**P**	A	A
*Ochroconis* cf. *musae*	S	A	**P**	A
*Paecilomyces* cf. *formosus*	S	A	**P**	**P**
*Paraconiothyrium archidendri*	S	A	**P**	A
*Paraphaeosphaeria* sp.		A	**P**	A
*Penicillium citrinum*		**P**	**P**	**P**
*Penicillium guaibinense*	S	A	**P**	A
*Penicillium* sp. 1 section *Lanata-Divaricata*		**P**	A	A
*Penicillium* sp. 2 section *Lanata-Divaricata*		A	A	**P**
*Penicillium* sp. 3 section *Brevicompacta*		A	A	**P**
*Phaeosphaeria musae*	S	**P**	A	A
*Polyschema* sp.	G	A	**P**	A
*Purpureocillium* cf. *lilacinum*		A	**P**	A
*Rhinocladiella similis*	S	A	**P**	A
*Sarocladium terricola*	S	**P**	A	A
*Talaromyces allahabadensis*	S	**P**	**P**	**P**
*Talaromyces* sp. 1 section *Talaromyces*		A	**P**	A
*Talaromyces* sp. 2 section *Talaromyces*		A	**P**	A
**Basidiomycota**				
“*Chondrostereum* sp.”	G	A	**P**	A
*Irpex* cf. *lacteus*	G, S	**P**	A	A
*Kwoniella* cf. *dendrophila*	G, S	A	**P**	A
*Rhodotorula* cf. *mucilaginosa*		A	**P**	A
“*Rigidoporus* sp.”		A	A	**P**
*Sakaguchia* sp.	G	A	**P**	A
*Schizophyllum commune*		**P**	A	A
*Trametes villosa*	S	**P**	A	A
**Mucoromycota**				
*Rhizopus arrhizus*		A	**P**	A
**Richness**		23	36	10

^a^ G = genus and S = species first record in a cave environment.

P = fungal taxon present (observed).

A = fungal taxon absent (not observed).

### Airborne fungi

From the samples collected to study airborne fungi we obtained 673 CFU, (527 using BHI and 146 using DRBC culture media). Sampling point 4 had the highest fungal abundance (F = 8.13 at 1% probability) (Fig 3A, S2 Table). Considering the sampling points defined from the cave entrance, point 2 presented a fungal richness of 11 taxa, followed by point 1 (10 taxa), point 3 (four taxa), and point 4 (two taxa). With five taxa, *Aspergillus* was the most representative genus in Ascomycota (mainly reported at point 1), and each genus in Basidiomycota was represented by one taxon (recorded at points 1, 2, and 3). Other genera commonly reported as airborne, such as *Cladosporium* and *Penicillium*, were also obtained at points 1 and 3 (S3 Table). At points 2, 3, and 4 we found *Aplosporella* sp., *Diaporthe* sp., and *Neodidymella* sp., which have been mainly reported in association with plant species, and *Beauveria bassiana*, a species commonly recorded as an entomopathogenic fungus. Isolates belonging to *Aplosporella*, *Neodidymella*, and *Nothophoma*, which have never previously been reported in caves, were also found to be airborne in this cave (Table 1).

### Bat fungi

**Fig 7:**
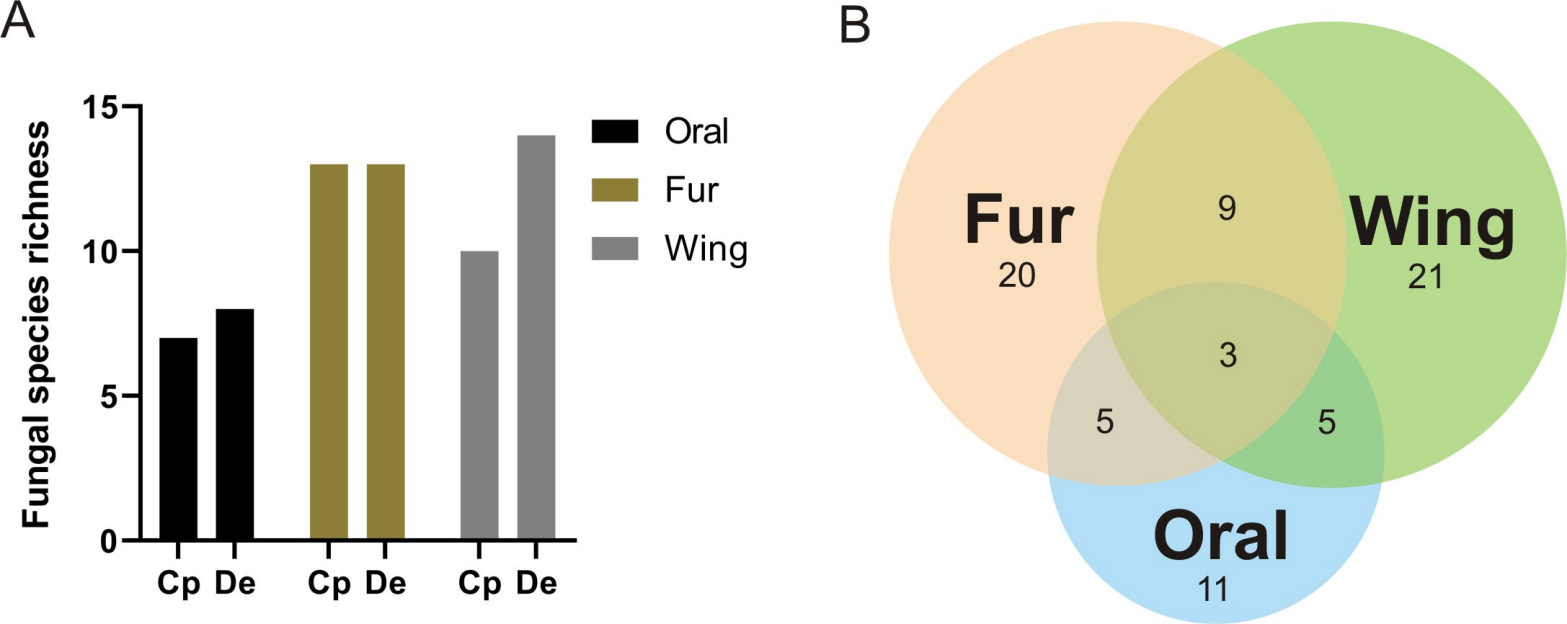
Fungal communities in the different microhabitats on the body of bats. Fungal richness (A) and taxa distribution (B) associated with different microhabitats (oral cavity, fur, and wing membrane) of the bats *Carollia perspicillata* (Cp) and *Diphylla ecaudata* (De) in the *Meu Rei* bat cave, Catimbau National Park, Pernambuco state, North-eastern region, Brazil.

All the seven captured bats showed fungal associations, with the vampire bat *D*. *ecaudata* showing a greater fungal abundance. The wing membrane had the largest number of fungi (45 CFU of 21 taxa, of which nine were exclusive), followed by the bat fur (35 CFU of 20 taxa, of which nine were exclusive), and the oral cavity (25 CFU of 11 taxa, of which four were exclusive) (Fig 3B, S4 Table). Interestingly, it was observed that the fungal richness was greater on the body and wing membrane than in the oral cavity of the two bat species. A pattern of fungal richness was observed for the oral cavity of the two bat species (seven from *C*. *perspicillata* and eight from *D*. *ecaudata*), with thirteen taxa on the fur of the two bat species, and ten taxa from *C*. *perspicillata* and 14 from *D*. *ecaudata* on the wing membrane. However, the fungal richness differed on the body and on the wing membrane between bat species. We found 21 exclusive fungal taxa on the wing membrane, followed by 20 on the fur, and 11 in the oral cavity; nine taxa were shared by the fur and the wing membrane, five taxa were shared by the wing membrane and the oral cavity, and the other five taxa by the oral cavity and the fur; three fungal taxa were shared by all three body microhabitats (Fig 7, S4 Table).

Analysis of fungal taxa in *C*. *perspicillata* and *D*. *ecaudata* together showed a difference between the mycobiota associated with the three different body microhabitats (*H* = 6.687, *P* = 0.035), suggesting that certain fungal species occur in specific areas. Analysis of the fungal composition of the microhabitats separately for each bat species, showed no significant difference for either *D*. *ecaudata* (*H* = 5.806, *P* = 0.063) or *C*. *perspicillata* (*H* = 2.024, *P* = 0.363) (S5 Table). In addition, the fungal species richness found in oral cavities, fur, or wings did not differ significantly between *C*. *perspicillata* and *D*. *ecaudata* (see S6 Table for details).

**Fig 8:**
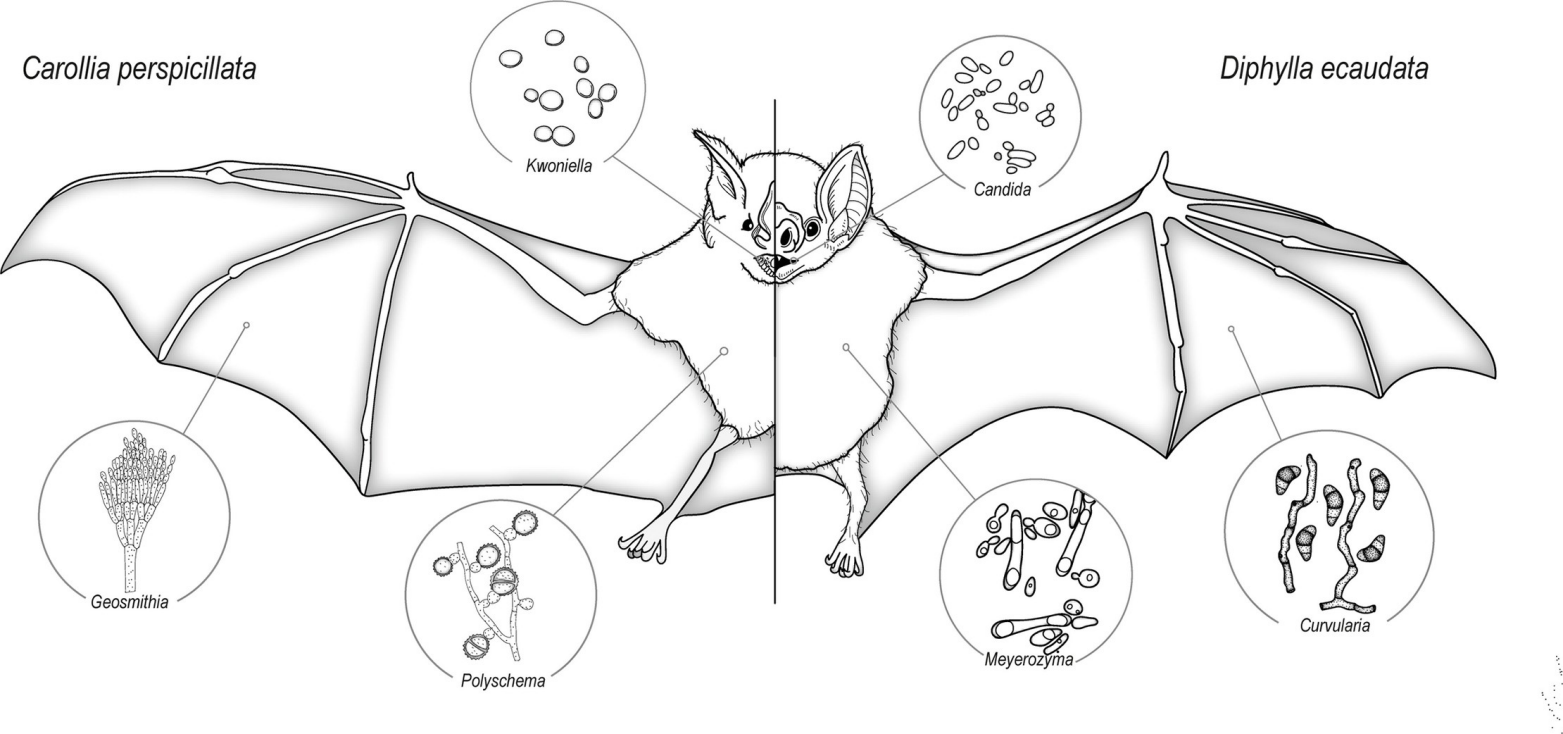
Fungal genera that were found on bats. Selected fungal genera exclusively associated with different microhabitats (oral cavity, fur, and wing membrane) of the bats *Carollia perspicillata* and *Diphylla ecaudata* in the *Meu Rei* bat cave, Catimbau National Park, Pernambuco state, North-eastern region, Brazil. Fungal illustrations were mainly redrawn from the book “The Genera of Hyphomycetes”.

The yeasts *Candida* spp. were isolated from all three bat microhabitats that we studied, and *Rhodotorula* cf. *mucilaginosa* was found in the oral cavity and on wing membranes (three isolates from each microhabitat). *Aspergillus* was the most representative genus (seven taxa) and the bat fur had four of these taxa. The unique Mucoromycota species, *Rhizopus arrhizus*, was found on the fur of *C*. *perspicillata* and on the wing membrane of *D*. *ecaudata*. Five fungal taxa found on the bat’s body were also observed as airborne and four others were also isolated from guano. Also isolated from the bats were: *Deniquelata quercina* and *Rhinocladiella similis* on *D*. *ecaudata*; *Fusarium* sp. *F*. *fujikuroi* complex and *Ochroconis* cf. *musae* (which are commonly found as plant pathogens, as endophytes, saprobes, or opportunistic pathogens) on *C*. *perspicillata* and *D*. *ecaudata*; *Paecilomyces* cf. *formosus* and *Purpureocillium* cf. *lilacinum* (which are reported as thermophiles or as entomopathogens) on *D*. *ecaudata*; and *Purpureocillium* cf. *lilacinum* on *C*. *perspicillata* and *D*. *ecaudata* (Fig 8). Similar to airborne samples, bats had six fungal taxa—*D*. *quercina*, *Nothophoma* sp., *Polyschema* sp., “*Chondrostereum* sp.”, *Kwoniella* cf. *dendrophila*, and *Sakaguchia* sp.—which are here reported from a cave environment for the first time (Table 1).

### Guano fungi

Guano had a total fungal abundance of 683 CFU; the guano of frugivorous species (CFU 56) was significantly different from the guano of insectivorous (CFU 368) and haematophagous species (CFU 259), which did not differ from each other significantly (F = 16.93 at 1% probability) (Fig 3C, S2 Table). The fungal richness did not differ greatly between insectivorous, frugivorous, and haematophagous bats or between fresh and non-fresh guano. A total of 10 taxa were identified, of which five were exclusively found only in the guano (Table 1). The guano of insectivorous and frugivorous bats had two exclusive taxa each (*Aspergillus* sp. 4 section *Polypaecilum* and *Paecilomyces* cf. *formosus* in the insectivorous bat guano and *A*. *sydowii* and *Humicola* cf. *seminuda* in the frugivorous bat guano), while haematophagous bats had one (*Penicillium citrinum*). Interestingly, *Aspergillus* sp. 4 section *Polypaecilum*, *P*. cf. *formosus*, and “*Rigidoporus* sp.” were isolated from guano of insectivorous bats, while *Aspergillus* and *Penicillium* were the main taxa isolated from the other guano samples (S7 Table).

## Discussion

Worldwide, few mycological studies have been undertaken in bat caves, and there is a gap in our knowledge of this mycobiome in tropical and subtropical countries [5, 18]. Our study indicated a high fungal species richness and diversity associated with a bat cave in Brazil’s Caatinga drylands. The airborne speleomycology of the *Meu Rei* bat cave in Brazil was estimated at four sampling points at increasing distances from the cave entrance, and we found that the fungal abundance was lower near the cave entrance and increased inwards to the fourth point (135 m from the cave entrance), where *A*. *geoffroyi*, *N*. *macrourus*, *P*. *gymnonotus*, and *P*. *personatus* were found in abundance. In contrast, the fungal richness was higher near the cave entrance and decreased towards the interior of the cave.

In a Brazilian cave, the number of terrestrial filamentous fungi was found to be higher deeper inside than at the entrance of the cave [30]. Similar results for airborne fungi were found in an active gold mine in South Africa [60]. Our findings are not consistent with studies which showed that the fungal abundance was higher near the entrance than that in the interior of a cave, but they agree with the higher fungal richness near the cave entrance [e.g. 5, 27, 61]. Thus, in our study, the higher fungal richness at the cave entrance may be the result of fungal material being transported in the wind; however, deeper inside the cave, material transported solely by the wind would not be able to reach the most distant parts, and bats would then become the main dispersal agent. Considering that the largest bat colonies are located in the deepest parts of the *Meu Rei* cave, this could explain the higher fungal densities that were observed there.

Studying a bat reserve in Poland with nearly 38,000 bats from 12 species, Kokurewicz et al. [11] found that more airborne fungi were isolated from inside the underground corridors than from the external environment. These authors also showed that the fungal concentration increased with increasing numbers of bats in the cave, showing that bats are the primary factor that determines the number of airborne fungi in hibernation sites [11]. Other authors have also highlighted that the mycobiome in caves may be influenced by the presence of bats that are responsible for fungal dispersion [5, 13, 22, 62].

The most commonly found airborne fungi in caves worldwide (*Aspergillus*, *Penicillium*, and *Cladosporium*) were also found in the *Meu Rei* bat cave. Species belonging to these genera have commonly been found in speleomycological studies in European, tropical, and subtropical countries [5]. For example, *Penicillium urticae* (currently *P*. *griseofulvum*) was the most frequently found species in a cave in Slovakia [20]. Similar results were found in Poland (where, in addition, *Cladosporium* was one of the most isolated fungi) [11], in a show cave in Spain [63], and from karst caves in China [16]. In Brazil, *Aspergillus* and *Penicillium* were the most abundant fungi found in different substrates of caves (e.g. soil and dry sediment) [21, 27, 29–31]. Other fungal taxa also reported in association with plants or as entomopathogenic fungus were also found as airborne fungi in our study [e.g. 5, 13, 16, 18].

The fungal abundance and richness was distinctive for each bat microhabitat, with the wing membrane showing higher values for both. Fur and skin of live bats can harbour a surprisingly higher fungal diversity than cave soils, which may be explained by the movement of bats between the surface and subterranean environments [5, 13, 64]. In the USA, wings of 30 bats from five bat hibernacula were found to be dominated by *Cladosporium*, *Fusarium*, *Geomyces*, *Mortierella*, *Penicillium*, and *Trichosporon*; *Geomyces* isolates were mainly obtained from damaged wings [62]. Johnson et al. [62] highlighted that fungi found on bat wings rather than those actively growing in this environment were an important reservoir of fungal spores in caves. The authors also suggested that some of these fungi, mainly *Geomyces* species, may act as minor pathogens living on bat wings.

The patterns of fungal species distribution on bats that we recorded in our study have also been observed in other studies. A large number of fungi commonly reported to be free-living in the environment or as being associated with plants were also found on the skin and fur of two subspecies of bent-winged bats in Australia [22]. The authors also showed that although all the bats that were examined carried fungi, the fungal community of some bats was dominated by a single species (e.g. *Aspergillus flavus*, *Rhodotorula mucilaginosa*, *Sporobolomyces roseus*, or *Ulocladium chartarum*). Similar to our work, species of yeasts were commonly isolated from bats and *R*. *mucilaginosa* was the most frequently isolated species in Australia [22]. New species were also described from bats; for example, a new cold-tolerant *Malassezia* species was isolated from the skin of bats in the subfamily Myotinae [23], and we also described a new *Geosmithia* species isolated from wing membrane of the frugivore bat *C*. *perspicillata* [65].

The role of the guano deposits produced by bats in harbouring fungi is also an important aspect to consider. Non-fresh insectivorous and haematophagous guano had the highest number of CFUs, whereas the fresh frugivorous guano had the lowest number. Bat guano is one of the most important reservoirs of nutrients in the cave environment and it is also a valuable source for invertebrate communities that may eat it directly or feed on the fungal species growing on the guano piles [5, 66, 67]. The origin (frugivorous, insectivorous, or haematophagous bats) and type (fresh and non-fresh) of the guano may also influence its composition (e.g. carbon, nitrogen, phosphorous, and polysaccharides) and pH [67–69]. Although, in general, fresh guano is basic (pH 8.5–9.0) and non-fresh guano is acidic (pH 5.0–5.5), the pH may vary with the deposition volume of urine, and with the age and depth of guano piles [67–69]. Some studies have highlighted that bat guano may also affect the fungal abundance and richness in caves because it can act as a common organic source for fungi and other organisms such as invertebrates [5, 20, 70].

The number of fungi isolated can vary because of the point of collection, and the origin and type of guano. In a show cave in Brazil, the largest number of fungal colonies was found in a fresh mixed guano deposition rather than as airborne [21] and a similar amount was found from non-fresh bat guano in two Slovakian caves [71]. An interesting result was obtained in Romania where, in caves with 3–10 bat species (varying from 200–7,000 individuals), a maximum number of fungi were obtained from bat guano [70]. In our study, the difference in fungal abundance in fresh and non-fresh insectivorous, frugivorous, and haematophagous guanos is an interesting finding which could help future studies identify and use culture media with distinctive composition and pH to recover a larger number of fungi. Fungal species can also require special compounds (e.g. carbon source) and a specific incubation temperature to grow in artificial conditions. Different methods have been used to isolate or to observe fungi living in guano samples, and these may also influence the observation of culturable fungal abundance and richness [16, 21, 70–72]. Metagenomic methods can also help in the study of fungal diversity in caves [e.g. 12, 22, 61, 73].

*Aspergillus* and *Penicillium* were the most common fungal isolates in bat guano. Species of *Aspergillus* and *Penicillium* have commonly been found in bat guano in Puerto Rico [72], Brazil [21], and Slovakia [71] along with other species of *Cladosporium*, *Purpureocillium*, *Trichoderma*, and *Xylaria*. In our study, records of *Aspergillus* sp. 4 section *Polypaecilum*, *Paecilomyces* cf. *formosus* (Ascomycota), and “*Rigidoporus* sp.” (Basidiomycota) in insectivorous bat guano are noteworthy, showing that the guano composition may influence and determine fungal growth. Species identified in *Aspergillus* section *Polypaecilum* are mainly treated as xerotolerant/xerophilic and halotolerant/halophilic and found in built environments [74], although a species was recently described from marine sediments in Mexico [75]. Species of *Paecilomyces* were found in acidic habitats and can tolerate microaerophilic conditions; however, *P*. *formosus* can also be found causing plant diseases and as an opportunistic pathogen in humans [76–78]. Basidiomycota taxa needs substrates rich in nutrients (such as wood and dung) to grow in cave environments, and species included in *Rigidoporus* are mainly found as plant pathogens [5, 72, 79]. Interestingly, new species of *Amphichorda*, *Gymnoascus*, and *Microascus* were found on bat guano in China [16], and as in our study, none of these studies reported the presence of *Histoplasma* associated with bat guano.

### Fungal diversity and bat cave conservation

Caves like *Meu Rei* are exceptional bat roosts, frequently harbouring high bat species richness. This is particularly true in the Caatinga dry forest in Brazil [2, 4]. Here, for the first time in Brazil, the speleomycology of a bat cave has been elucidated, revealing a remarkable diversity. Eight (21.6%) of the 37 genera (Ascomycota = *Aplosporella*, *Deniquelata*, *Neodidymella*, *Nothophoma*, and *Polyschema* and Basidiomycota = “*Chondrostereum*”, *Kwoniella*, and *Sakaguchia*) and 17 (53.1%) of the 32 identified species are reported for the first time from caves worldwide, and all fungi discovered in this study are formally recorded for the first time in a bat cave. In the world review of cave fungi, Vanderwolf et al. [5] listed 1,029 species from 518 genera and showed that approximately 59% of the taxa were reported from a single location, and many species were rarely isolated. Subsequently, other studies added more fungal taxa to that list, increasing the number of fungi found in caves [e.g. 11, 15–18, 20–27, 64, 71, 80, 81]. In two karst caves in China, Zhang et al. [16] reported that 28 (24%) of the 116 genera and 111 (59%) of the 188 identified species were reported for the first time in cave environments. Subsequently, Zhang et al. [18] described 33 new fungal species and found about 30% of the genera and 53% of the species for the first time in karst caves in China. Thus, the known number of fungal species documented from cave environments has increased to nearly 2,000. Such examples highlight the large potential such sites may have to harbour as yet unknown fungal species.

Our study of the culturable mycobiome in a Brazilian bat cave revealed a remarkable fungal diversity, including unknown taxa in cave environments and new species that will be described in further detail elsewhere. The fungal diversity in caves has a distinctive distribution pattern which can be influenced by cave apertures, air current, bat presence, and guano deposition, among other factors; however, several common species found in these environments are also reported from outside the cave, mainly in association with plants and soil. As shown by Zhang et al. [7], fungal communities in these locations are from land surfaces and common fungal species found in that biome are explained as ‘the geographic history of caves appeared to be too short for fungal speciation’. The inclusion of methods to study the non-culturable fungal diversity in caves will also help in the recovery of fungi that remain undetected in these subterranean environments. Further studies are being conducted in other caves in Brazil’s Caatinga drylands, which will help in the understanding of fungal community composition, contribute to national and global fungal estimation, and augment appeals for the preservation of the Caatinga.

## Supporting information

S1 Fig Phylogenetic diversity of filamentous fungal isolates using *TUB2* sequences. (TIF)

S1 Table GenBank accession numbers. (XLS)

S2 Table General number of fungal colonies (CFU). (DOC)

S3 Table Airborne fungi. (DOC)

S4 Table Bat fungi. (DOC)

S5 Table Differences in fungal species richness in bats. (DOC)

S6 Table Comparison of fungal species composition in bat microhabitats and species. (DOC)

S7 Table Guano fungi. (DOC)
