# Supplementary figures and images for: Living in the dark: Bat caves as hotspots of fungal diversity
Source: PLoS One. 2020 Dec 4;15(12):e0243494. doi: 10.1371/journal.pone.0243494 (PMC7717564; doi:10.1371/journal.pone.0243494)

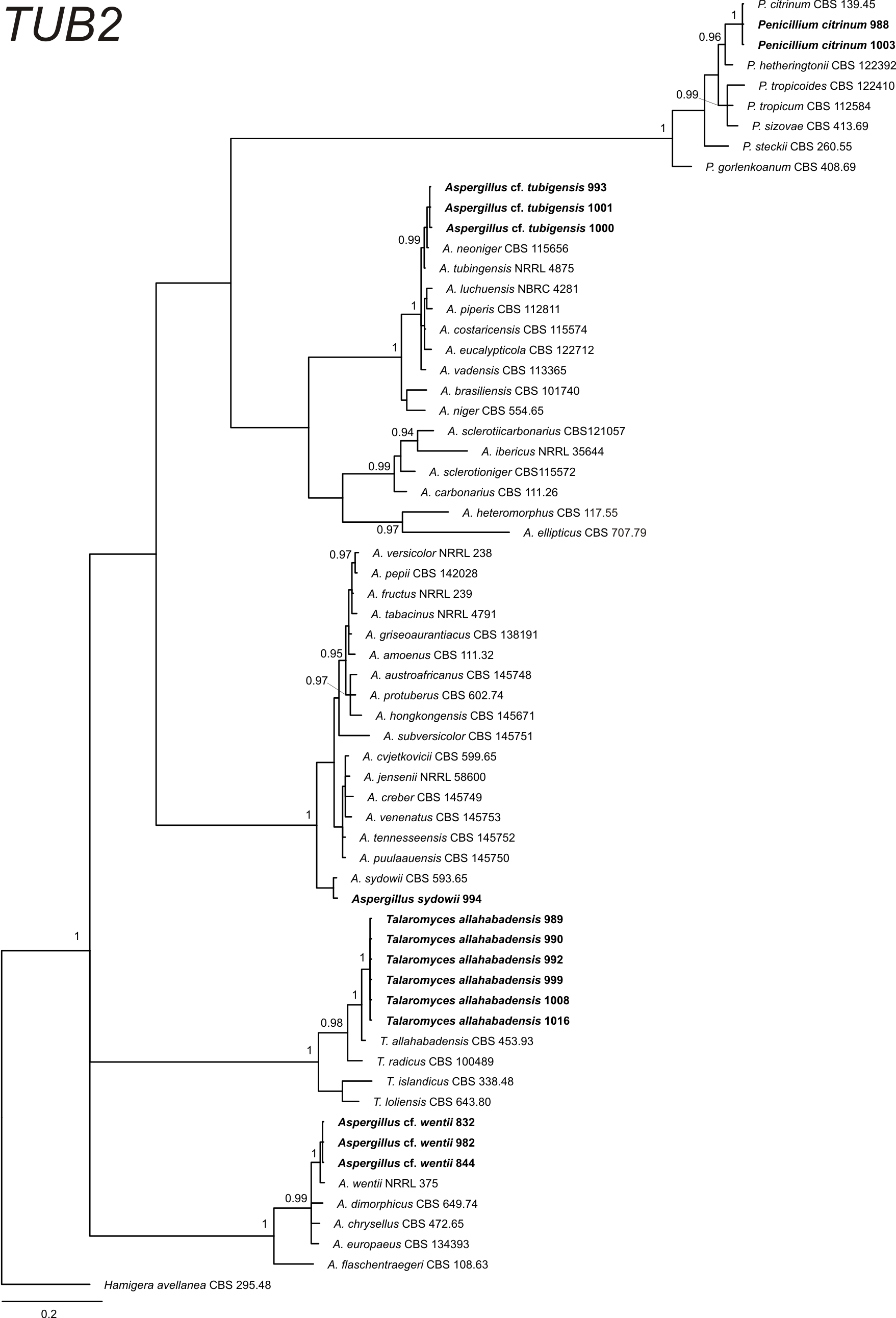

Supplement: S1 Fig — (TIF) [file pone.0243494.s001.tif]
