# Supplementary material for: Living in the dark: Bat caves as hotspots of fungal diversity
Source: PLoS One. 2020 Dec 4;15(12):e0243494. doi: 10.1371/journal.pone.0243494 (PMC7717564; doi:10.1371/journal.pone.0243494)
Supplement: S2 Table — (DOC) [file pone.0243494.s003.doc]

**S2 Table. General number of fungal colonies (CFU).** Fungal abundance of airborne, bat, and guano represented by the total number of colonies (CFU) observed during the fieldwork in the *Meu Rei* bat cave, Catimbau National Park, Caatinga dry forest, Pernambuco state, North-eastern region of Brazil.

| **Airborne** | **CFU** | | |
| --- | --- | --- | --- |
| Point 1 | 49 | | |
| Point 2 | 51 | | |
| Point 3 | 121 | | |
| Point 4 | 452 | | |
| **Bat** | **Cp** | **De** | **Total** |
| Oral cavity | 12 | 13 | 25 |
| Fur | 16 | 19 | 35 |
| Membrane wings | 17 | 28 | 45 |
| **Guano** | **F** | **NF** | **Total** |
| Insectivorous | 162 | 206 | 368 |
| Frugivorous | 42 | 14 | 56 |
| Hematophagous | 46 | 213 | 259 |

Cp = *Carollia perspicillata* and De = *Diphylla ecaudata*.

F = fresh and NF = non-fresh guano.
