# Supplementary material for: Living in the dark: Bat caves as hotspots of fungal diversity
Source: PLoS One. 2020 Dec 4;15(12):e0243494. doi: 10.1371/journal.pone.0243494 (PMC7717564; doi:10.1371/journal.pone.0243494)
Supplement: S3 Table — (DOC) [file pone.0243494.s004.doc]

**S3 Table. Airborne fungi.** Richness of airborne fungi isolated in the *Meu Rei* bat cave located at the Catimbau National Park, Caatinga dry forest, Pernambuco state, North-eastern region of Brazil. Points means the distance from the cave entrance: 15 m (point 1), 45 m (point 2), 75 m (point 3), and 135 m (point 4).

| **Fungi** | **Point 1** | **Point 2** | **Point 3** | **Point 4** |
| --- | --- | --- | --- | --- |
| **Ascomycota** |  |  |  |  |
| *Aplosporella* sp. | A | A | A | **P** |
| *Aspergillus bertholletiae* | **P** | A | A | A |
| *Aspergillus* cf. *sesamicola* | **P** | A | A | A |
| *Aspergillus* cf. *wentii* | **P** | A | A | A |
| *Aspergillus ochraceus* | **P** | A | A | **P** |
| *Aspergillus sydowii* | **P** | A | A | A |
| *Beauveria bassiana* | A | A | **P** | A |
| *Cladosporium* sp. 1 *C. sphaerospermum* complex | **P** | A | A | A |
| *Cladosporium* sp. 3 *C. cladosporioides* complex | A | A | **P** | A |
| *Diaporthe* sp. 1 | A | **P** | A | A |
| *Diaporthe* sp. 2 | A | **P** | A | A |
| *Humicola* cf. *seminuda* | **P** | **P** | A | A |
| *Myceliophthora* sp. | A | **P** | A | A |
| *Neodidymella thailandicum* | A | **P** | A | A |
| *Nothophoma* sp. 2 | A | **P** | A | A |
| *Penicillium citrinum* | A | A | **P** | A |
| *Penicillium* sp. 1 section *Lanata-Divaricata* | **P** | A | A | A |
| *Phaeosphaeria musae* | A | **P** | A | A |
| *Sarocladium terricola* | A | **P** | A | A |
| *Talaromyces allahabadensis* | A | **P** | A | A |
| **Basidiomycota** |  |  |  |  |
| *Irpex* cf. *lacteus* | A | **P** | A | A |
| *Schizophyllum commune* | **P** | **P** | **P** | A |
| *Trametes villosa* | **P** | A | A | A |
| **Richness** | 10 | 11 | 4 | 2 |

P = fungal taxon present (observed).

A = fungal taxon absent (not observed).
