# Supplementary material for: Living in the dark: Bat caves as hotspots of fungal diversity
Source: PLoS One. 2020 Dec 4;15(12):e0243494. doi: 10.1371/journal.pone.0243494 (PMC7717564; doi:10.1371/journal.pone.0243494)
Supplement: S4 Table — (DOC) [file pone.0243494.s005.doc]

**S4 Table. Bat fungi.** Richness and abundance of fungi isolated from the oral cavity, fur, and membrane wings of two bats species (*Carollia perspicillata* [Cp] and *Diphylla ecaudata* [De]) in the *Meu Rei* bat cave located at the Catimbau National Park, Caatinga dry forest, Pernambuco state, North-eastern region of Brazil.

| **Fungi** | **Oral cavity** | | **Fur** | | **Membrane wings** | |
| --- | --- | --- | --- | --- | --- | --- |
|  | Cp | De | Cp | De | Cp | De |
| **Ascomycota** |  |  |  |  |  |  |
| *Aspergillus* cf. *wentii* |  |  |  | 1 |  |  |
| *Aspergillus* sp. 1 section *Nidulantes* |  | 1 |  |  |  |  |
| *Aspergillus* sp. 2 section *Nigri* |  |  |  | 1 |  |  |
| *Aspergillus* sp. 3 section *Nigri* |  |  |  |  |  | 2 |
| *Aspergillus* sp. 5 section *Aspergillus* |  |  |  | 1 |  |  |
| *Aspergillus sydowii* |  |  |  | 1 |  |  |
| *Aspergillus westerdijkiae* |  | 1 |  |  |  |  |
| *Candida orthopsilosis* | 3 | 1 | 1 |  |  |  |
| *Candida parapsilosis* | 2 | 3 | 1 | 2 | 3 |  |
| *Cladosporium* sp. 2 *C. sphaerospermum* complex |  |  |  |  | 1 |  |
| *Cladosporium* sp. 3 *C. cladosporioides* complex | 1 | 3 | 1 | 1 | 1 | 4 |
| *Curvularia* sp. |  |  |  |  |  | 1 |
| *Deniquelata quercina* |  |  |  |  |  | 1 |
| *Fusarium* sp. *F. fujikuroi* complex | 1 | 2 | 1 |  | 1 |  |
| *Geosmithia carolliae* |  |  |  |  | 3 |  |
| *Gymnoascus dankaliensis* |  |  |  |  |  | 1 |
| *Hypoxylon* sp. |  |  | 1 |  |  |  |
| *Meyerozyma* cf. *caribbica* |  |  |  | 2 |  |  |
| *Nothophoma* sp. 1 |  |  |  | 1 |  | 1 |
| *Ochroconis* cf. *musae* |  | 1 | 1 | 1 |  |  |
| *Paecilomyces* cf. *formosus* |  |  |  | 1 |  | 5 |
| *Paraconiothyrium archidendri* |  |  |  |  | 1 |  |
| *Paraphaerosphaeria* sp. | 1 |  |  |  |  |  |
| *Penicillium citrinum* |  | 1 |  |  | 2 |  |
| *Penicillium guaibinense* |  |  | 1 |  |  |  |
| *Polyschema* sp. |  |  | 1 |  |  |  |
| *Purpureocillium* cf. *lilacinum* |  |  | 1 | 4 | 2 | 3 |
| *Rhinocladiella similis* |  |  |  |  |  | 1 |
| *Talaromyces allahabadensis* |  |  | 3 | 1 |  | 6 |
| *Talaromyces* sp. 1 section *Talaromyces* |  |  | 1 |  |  |  |
| *Talaromyces* sp. 2 section *Talaromyces* |  |  | 1 | 2 |  | 1 |
| **Basidiomycota** |  |  |  |  |  |  |
| “*Chondrostereum* sp.” |  |  |  |  |  | 1 |
| *Kwoniella* cf. *dendrophila* | 1 |  |  |  |  |  |
| *Rhodotorula* cf. *mucilaginosa* | 3 |  |  |  | 2 | 1 |
| *Sakaguchia* sp. |  |  |  |  | 1 |  |
| **Mucoromycota** |  |  |  |  |  |  |
| *Rhizopus arrhizus* |  |  | 1 |  |  | 1 |
| **Number of isolates** | 12 | 13 | 15 | 19 | 17 | 29 |
| **Richness** | 7 | 8 | 13 | 13 | 10 | 14 |
