# Supplementary material for: Living in the dark: Bat caves as hotspots of fungal diversity
Source: PLoS One. 2020 Dec 4;15(12):e0243494. doi: 10.1371/journal.pone.0243494 (PMC7717564; doi:10.1371/journal.pone.0243494)
Supplement: S5 Table — (DOC) [file pone.0243494.s006.doc]

**S5 Table. Differences in fungal species richness in bats.** Kruskal-Wallis test and the post-hoc Dunn’s pairwise test showing the significance of the difference in fungal composition in different microhabitat of the bat’s body (*Carollia perspicillata* and *Diphylla ecaudata*).

| **Attribute** | **Kruskal-Wallis test** | | **Dunn’s pairwise comparison test** | | | | | |
| --- | --- | --- | --- | --- | --- | --- | --- | --- |
|  |  |  | **Fur *vs.* Oral** | | **Fur *vs.* Wing** | | **Oral *vs.* Wing** | |
| Fur *vs.* Oral *vs.* Wing  (both bat species) | *H* | *P* | Q | *P* | Q | *P* | Q | *P* |
|  | 6.687 | **0.035** | 1.829 | **0.034** | 0.203 | 0.814 | 2.03 | **0.018** |
|  |  |  | **Fur *vs.* Oral** | | **Fur *vs.* Wing** | | **Wing *vs.* Oral** | |
| Fur *vs.* Oral *vs.* Wing  (*D*. *ecaudata*) | *H* | *P* | Q | *P* | Q | *P* | Q | *P* |
|  | 5.806 | 0.063 | 1.693 | 0.090 | 0.564 | 0.572 | 2.257 | **0.023** |
|  |  |  | **Fur *vs.* Oral** | | **Fur *vs.* Wing** | | **Wing *vs.* Oral** | |
| Fur *vs.* Oral *vs.* Wing  (*C*. *perspicillata*) | *H* | *P* | Q | *P* | Q | *P* | Q | *P* |
|  | 2.024 | 0.363 | 1.232 | 0.217 | 0 | 1 | 1.232 | 0.217 |
