# Supplementary material for: Living in the dark: Bat caves as hotspots of fungal diversity
Source: PLoS One. 2020 Dec 4;15(12):e0243494. doi: 10.1371/journal.pone.0243494 (PMC7717564; doi:10.1371/journal.pone.0243494)
Supplement: S6 Table — (DOC) [file pone.0243494.s007.doc]

**S6 Table. Comparison of fungal species composition in bat microhabitats and species.** Comparison of the fungal composition between *Carollia perspicillata* and *Diphylla ecaudata* (Chiroptera) found on different parts of their bodies.

| **Body’s microhabitat** | **Fungus species richness** | | **Mann-Whitney test** | |
| --- | --- | --- | --- | --- |
|  | *C*. *perspicillata* | *D*. *ecaudata* | *U* | *P* |
| Fur | 13 | 16 | 272.000 | 0.990 |
| Oral cavity | 9 | 9 | 250.500 | 0.576 |
| Wing | 13 | 17 | 263.500 | 0.817 |
