# Supplementary material for: Living in the dark: Bat caves as hotspots of fungal diversity
Source: PLoS One. 2020 Dec 4;15(12):e0243494. doi: 10.1371/journal.pone.0243494 (PMC7717564; doi:10.1371/journal.pone.0243494)
Supplement: S7 Table — (DOC) [file pone.0243494.s008.doc]

**S7 Table. Guano fungi.** Richness of fungi isolated from fresh and non-fresh guano of insectivorous, frugivorous, and hematophagous bats in the *Meu Rei* bat cave located at the Catimbau National Park, Caatinga dry forest, Pernambuco state, North-eastern region of Brazil.

| **Fungi** | **Insectivorous** | | **Frugivorous** | | **Hematophagous** | |
| --- | --- | --- | --- | --- | --- | --- |
|  | Fresh | Not fresh | Fresh | Not fresh | Fresh | Not fresh |
| **Ascomycota** |  |  |  |  |  |  |
| *Aspergillus* cf. *tubingensis* | A | A | **P** | A | A | **P** |
| *Aspergillus* sp. 4 section *Polypaecilum* | **P** | **P** | A | A | A | A |
| *Aspergillus sydowii* | A | A | **P** | A | A | A |
| *Humicola* cf. *seminuda* | A | A | A | **P** | A | A |
| *Paecilomyces* cf. *formosus* | **P** | A | A | A | A | A |
| *Penicillium citrinum* | A | A | A | A | **P** | A |
| *Penicillium* sp. 2 section *Lanata-Divaricata* | **P** | **P** | A | A | **P** | A |
| *Talaromyces allahabadensis* | A | A | **P** | **P** | **P** | **P** |
| **Basidiomycota** |  |  |  |  |  |  |
| “*Rigidoporus* sp.” | A | **P** | A | A | A | A |
| **Richness** | 3 | 3 | 3 | 2 | 3 | 2 |

P = fungal taxon present (observed).

A = fungal taxon absent (not observed).
